# Comparative Outcomes of Adalimumab and Infliximab Dose Escalation in Inflammatory Bowel Disease Patients Failing First-Line Biologic Treatment

**DOI:** 10.3390/jcm14041228

**Published:** 2025-02-13

**Authors:** Ali Atay, Yavuz Cagir, Mucahit Ergul, Oguz Ozturk, Muhammed Bahaddin Durak, Ilhami Yuksel

**Affiliations:** 1 Department of Gastroenterology, Ankara Bilkent City Hospital, 06800 Ankara, Turkey; dr.mucahitergul@gmail.com (M.E.); oguzozturk90@gmail.com (O.O.); iyuksel@aybu.edu.tr (I.Y.); 2 Department of Gastroenterology, Ankara Yildirim Beyazit University Yenimahalle Training and Research Hospital, 06370 Ankara, Turkey; yvzcgr@hotmail.com; 3 Department of Gastroenterology, Faculty of Medicine, Hacettepe University, 06100 Ankara, Turkey; doctormbd@gmail.com; 4 Department of Gastroenterology, School of Medicine, Ankara Yildirim Beyazit University, 06800 Ankara, Turkey

**Keywords:** dose escalation, adalimumab, infliximab, inflammatory bowel disease, ulcerative colitis, Crohn’s disease

## Abstract

**Background/Objectives:** Dose escalation has been commonly used to achieve and maintain response. We aimed to compare the outcomes of adalimumab or infliximab dose escalation in inflammatory bowel disease (IBD) patients. **Methods:** Treatment persistence (TP) and predictive factors for remission-free treatment discontinuation (r-fTD) were evaluated in patients treated with adalimumab or infliximab dose escalation between 2019 and 2024. **Results:** Dose escalation was identified in 142 patients treated with adalimumab (UC: 23.9%; CD: 76.1%) and in 126 patients treated with infliximab (UC: 23.8%; CD: 76.2%). The TP rate was significantly lower in the adalimumab group (35.2%) than the infliximab group (53.2%) (*p* = 0.003). The survival analysis showed that drug persistence was lower in the adalimumab group compared with the infliximab group (mean time: 74.3 vs. 99.5 months, *p* < 0.001). TP rates showed no significant differences between UC and CD for both adalimumab (mean time UC: 64.7 months vs. CD: 76.2 months, *p* = 0.403) and infliximab (mean time UC: 80.3 months and CD: 102.6 months, *p* = 0.151). The r-fTD rates were significantly higher in the adalimumab group (62.7%) than the infliximab group (39.7%) (*p* < 0.001). Primary lack of response and secondary loss of response (sLOR) rates were both higher in the adalimumab group (7.7% and 51.4%) than the infliximab group (1.6% and 28.6%). However, serious adverse events were lower in the adalimumab group (2.1%) than the infliximab group (7.9%) (*p* = 0.027). **Conclusions:** Infliximab dose escalation was more effective than adalimumab in both UC and CD patients. Regarding the side effect profile, adalimumab dose escalation was found to be safer compared with infliximab.

## 1. Introduction

Inflammatory bowel disease (IBD) is a chronic inflammatory disease that includes Crohn’s disease (CD) and ulcerative colitis (UC) [1,2,3]. Drugs targeting tumor necrosis factor alpha (TNF-α) have revolutionized the treatment of IBD refractory to conventional treatments [4]. A significant proportion of the remaining patients experience secondary loss of response (sLOR) during anti-TNF-α maintenance therapy; the annual risk ranges from around 10% to 20%, and cumulative sLOR rates are reported to be as high as 60% over the course of treatment [4,5,6,7]. These scenarios present serious challenges for gastroenterologists managing IBD. Previous research demonstrated that sLOR to anti–TNF-α therapies can result from inadequate dosing and anti-drug antibodies [8,9]. Dose escalation has commonly been observed as a treatment strategy, even beyond regulatory recommendations, to achieve and maintain response, with average rates of dose escalation estimated at 30–36% in IBD patients over 1 year with anti-TNF-α or anti-integrin agents [10,11]. Since the concept of therapeutic dose monitoring has recently been introduced for ensuring dose titration of anti-TNFs, there is still a lack of consensus on whether therapeutic dose monitoring or the traditional dose escalation method should be adopted in cases of sLOR [12,13,14].

Although meta-analysis exists comparing the effects of adalimumab and infliximab dose escalation, no study has yet provided a comparative analysis of these therapies specifically in IBD patients who required dose escalation due to failure of first-line anti-TNF-α therapy. In this study, we aimed to evaluate the need for dose escalation and the treatment persistence (TP) rate in biologic-naïve patients with IBD receiving adalimumab or infliximab dose escalation, while also identifying the predictive factors influencing remission-free treatment discontinuation (r-fTD).

## 2. Material and Methods

### 2.1. Study Design and Patients

We performed an observational, retrospective, single-center study. All adult biologic-naïve patients with IBD who were treated with anti-TNF-α agents and received maintenance therapy for more than 4 months between July 2019 and July 2024 were assessed for inclusion in the study. These patients were diagnosed with IBD based on clinical symptoms, laboratory examination, imaging, endoscopy, and pathological findings according to internationally accepted criteria [12,15] and classified according to the Montreal classification system [16]. Patients with unclassified IBD types and biologic-naïve patients treated with certolizumab pegol and golimumab were excluded from the study due to the small sample sizes.

The treatment persistence and the predictive factors for r-fTD were evaluated in patients receiving anti-TNF-α agent dose escalation who experienced sLOR to the standardized dose. For those receiving maintenance treatment with adalimumab at a subcutaneous dose of 40 mg every 2 weeks, the dose interval had to be reduced to one week if dose escalation was required. In patients treated with infliximab at 5 mg/kg intravenously every 8 weeks, dose escalation to 10 mg/kg was accepted if dose escalation was required. Patients with less than 6 months of follow-up after the initiation of dose escalation were not included in the evaluation of treatment response to dose escalation. The stages of the study population formation are presented in a flow chart.

The data were collected from an online database system used for follow-up of patients with IBD. Data including age at diagnosis, gender, smoking history, family history of IBD, history of appendectomy, body mass index (BMI), total disease duration, time interval between IBD onset and starting biologic treatment, follow-up time from initiating biologic treatment to dose escalation time, follow-up time from dose escalation time to last visit, presence of extra-intestinal manifestations, and history of medical therapy or prior major abdominal surgery for IBD were collected. Laboratory analysis (C-reactive protein [CRP], hemoglobin, and albumin), and Crohn’s disease activity index (CDAI) for CD patients or partial and endoscopic Mayo score for UC patients were recorded at the initiation of dose escalation. In addition, history of conventional therapy as well as concomitant medications including 5-aminosalicylate, steroids and immunomodulators (thiopurine or methotrexate) were documented.

### 2.2. Variables and Outcomes

Treatment persistence was defined as the continued use of adalimumab or infliximab with dose escalation until the study’s termination. Treatment discontinuation was divided into sustained remission and r-fTD. The causes of r-fTD were classified as patient non-compliance, patient intolerance, and therapeutic failure. Therapeutic failure was further divided into primary lack of response (pLOR) and sLOR. sLOR included the need for corticosteroids, IBD-related hospitalization, IBD-related surgery, switching to another biologic therapy, and serious adverse events (allergic reactions, serious infections, or other significant adverse events). Pregnancy was not specifically highlighted in our study, as none of the patients became pregnant during the study period.

Clinical response was defined as the absence of abdominal pain and general well-being for CD, and the absence of rectal bleeding and normalization of defecation frequency for UC. Gastroenterologists assessed the biochemical and clinical response at each visit. The pLOR for adalimumab dose escalation was defined as discontinuation before week 14 due to treatment failure [17], whereas for infliximab dose escalation, it was defined as a lack of clinical response after induction therapy (week 0–2–6, 10 mg/kg, at least 2 infusions), leading to treatment discontinuation [18]. sLOR was defined as the recurrence of signs of disease activity after an initial response to treatment [7,19].

### 2.3. Statistical Analysis

The data were analyzed using IBM SPSS Statistics for Windows, version 27.0 (IBM Corp., Armonk, NY, USA). Categorical data were summarized using frequency (n) and percentage (%), while numerical data were presented as median values with interquartile range (IQR). The Pearson Chi-square test or Fisher’s exact test was used to compare categorical data. The Mann–Whitney U test was used to compare numerical data. Univariate Cox regression analysis was conducted to examine factors associated with TP across the entire study population, as well as separately for UC, CD, and all IBD patients receiving adalimumab or infliximab. Subsequently, multivariate Cox models were employed to identify the independent predictors of TP for anti-TNF-α agents. Variables with a *p*-value of <0.1 in the bivariate analysis were included in the multivariate models. To compare TP over the period from initiation of biologic agents to treatment discontinuation, Kaplan–Meier survival analysis was performed. Mantel–Cox log-rank testing was used to assess differences in TP rates between patient subgroups. In the survival analysis, discontinuation of medication was considered as the “event”, excluding the 12 patients who achieved “sustained remission” (n = 139). The time variable was defined as follows: the duration from the start of the medication to the date of discontinuation for patients with an “event”; the duration from the start of the medication to the achievement of remission for “sustained remission” patients; and the duration from the start of the medication to the last visit for other patients. A *p*-value of <0.05 was set as the threshold for statistical significance.

## 3. Results

### 3.1. Study Population, Demographics and Clinical Findings

A total of 1686 patients with IBD were initially considered, of whom 832 (49.3%) were treated with biologics and 854 (50.7%) received conventional therapy, and 83 patients were excluded from the study. The remaining 749 patients received adalimumab or infliximab as first-line biologic therapies. Of those, 409 (54.6%) were treated with adalimumab, and 340 (45.4%) with infliximab. A total of 268 (35.7%) patients experienced dose escalation, including 142 (34.7%) patients on adalimumab (UC: 34, 23.9% and CD: 108, 76.1%) and 126 (37.0%) patients on infliximab (UC: 30, 23.8% and CD: 96, 76.2%) (Supplementary Figure S1).

The demographics, clinical characteristics, and laboratory findings for the IBD patients receiving adalimumab and infliximab dose escalation therapies are summarized in Table 1. BMI was slightly higher in patients receiving adalimumab than in those receiving infliximab (*p* = 0.021). Erythema nodosum was significantly more prevalent in the adalimumab group than in the infliximab group (5.6% vs. 0.8%, *p* = 0.039). Patients in the adalimumab group had received budesonide therapy before biologic treatment at a statistically significantly higher frequency than in the infliximab group (28.2 vs. 11.9%, *p* = 0.001). Patients receiving adalimumab had a significantly lower rate of concomitant thiopurine use compared with those receiving infliximab (25.4% vs. 36.5%, *p* = 0.048). All other characteristics were similar between groups.

### 3.2. Outcomes of Treatments

The treatment persistence rate in patients receiving adalimumab was significantly lower compared with the infliximab group (35.2% vs. 53.2%), while the treatment discontinuation rate was significantly higher in the adalimumab group compared with the infliximab group (64.8% vs. 46.8%) (*p* = 0.003). The sustained remission rate in the adalimumab group was also significantly lower than in the infliximab group (2.1% vs. 7.1%, *p* = 0.047). Furthermore, the r-fTD rate in patients receiving adalimumab was significantly higher than in the infliximab group (62.7% vs. 39.7%, *p* < 0.001). Lack of compliance occurred at similar rates in the adalimumab and infliximab groups (1.4% vs. 1.6%, *p* = 0.999), while patient intolerance was significantly lower in the adalimumab group compared with the infliximab group (1.4% vs. 7.9%, *p* = 0.010). The therapeutic failure rate in patients receiving adalimumab was significantly higher than in the infliximab group (59.1% vs. 30.2%, *p* < 0.001). The causes of therapeutic failure were assessed; the need for steroid therapy and switches to other biologic therapies were significantly higher in the adalimumab group than in the infliximab group (10.6% vs. 3.6%, *p* = 0.019 and 50.7% vs. 27.8%, *p* < 0.001, respectively). However, the rate of serious adverse events was significantly lower in the adalimumab group compared with the infliximab group (2.1% vs. 7.9%, *p* = 0.027). Other causes of therapeutic failure were similar between the two groups. Allergic reactions were observed in 5.6% of patients in the infliximab group, but not in the adalimumab group (*p* = 0.005). The rates of serious infection and other serious adverse events were similar between the adalimumab and infliximab groups (2.1% vs. 0%, *p* = 0.250 and 0% vs. 2.4%, *p* = 0.103, respectively) (Table 2).

The rates of r-fTD in UC patients were similar between the adalimumab and infliximab groups (64.7% vs. 43.3%, *p* = 0.087). In contrast, r-fTD rates in CD patients was significantly higher in the adalimumab group compared with the infliximab group (62.0% vs. 38.5%, *p* = 0.001). No significant differences were observed in patient characteristics between UC and CD patients within either treatment group (Table 3).

### 3.3. Predictors of Remission-Free Treatment Discontinuation

The results of the bivariate and multivariate analysis of the predictors of r-fTD were evaluated. Upon adjusting for statistically significant factors from the bivariate analysis in the multivariate Cox regression model, smoking cessation (HR: 0.64 [95% CI: 0.43–0.94], *p* = 0.024) was associated with higher TP rates in both groups. However, prior major abdominal surgery for IBD (HR: 1.85 [95% CI: 1.29–2.67], *p* = 0.001), previous use of mesalazine enema (HR: 2.42 [95% CI: 1.59–3.66], prior use of methotrexate (HR: 1.83 [95% CI: 1.29–2.59], *p* = 0.001), and concomitant use of steroids (HR: 5.31 [95% CI: 2.22–12.68], *p* < 0.001) were associated with a higher r-fTD rate in the overall study population. In the multivariate analysis performed separately for both anti-TNF-α agents, being an ex-smoker (*p* = 0.033) was associated with a lower r-fTD rate in patients receiving adalimumab. Conversely, prior methotrexate use (*p* = 0.018), steroid dependence (*p* = 0.023), and concomitant steroid use (*p* = 0.003) were associated with a higher r-fTD rate in the adalimumab group. In the infliximab group, a family history of IBD (*p* = 0.012), prior major abdominal surgery for IBD (*p* = 0.004), and previous use of mesalazine enema (*p* < 0.001) were significantly associated with a higher r-fTD rate (Table 4).

### 3.4. Predictors by Disease Type

Disease extension in UC patients and disease location in CD patients were not associated with r-fTD in either group. In CD patients, disease penetration was associated with higher r-fTD in the infliximab group (*p* = 0.004), while in UC patients, baseline partial Mayo score correlated with r-fTD in the adalimumab group (*p* = 0.007) and baseline CDAI score in CD patients was associated only with the infliximab group (*p* = 0.017) in the bivariate analysis. Nevertheless, the multivariate analysis eliminated all the above variables (Table 5).

### 3.5. Survival Analysis of Treatments

**Figure 1:**
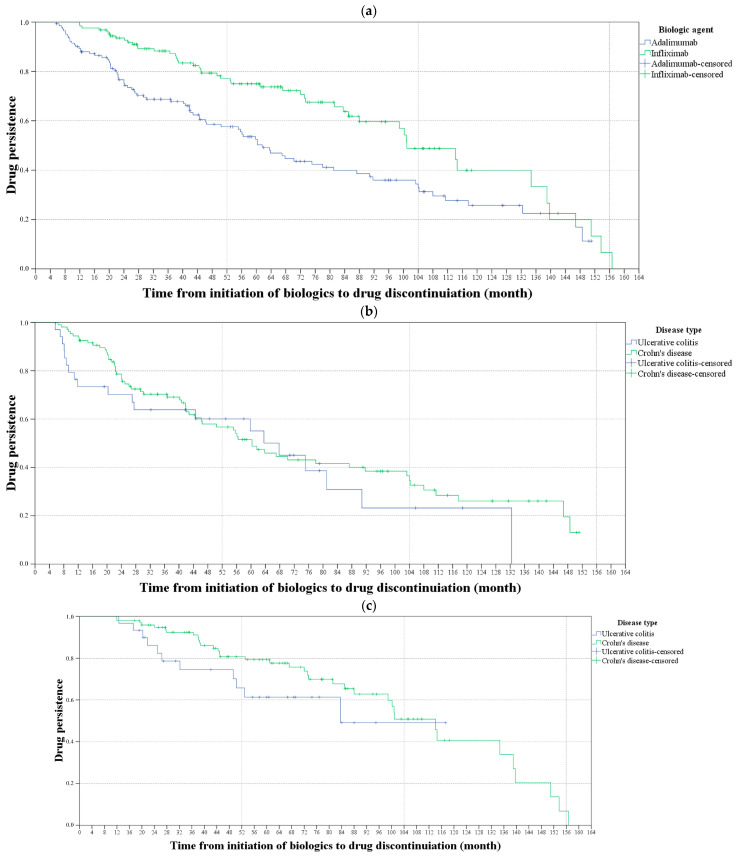
Kaplan–Meier survival analysis for evaluating drug persistence in patients with inflammatory bowel disease. (**a**) The blue line shows the drug persistence rates for the patients receiving adalimumab, and the green line shows the drug persistence rates for those receiving infliximab. The drug persistence rate was significantly higher in patients receiving infliximab than receiving adalimumab (*p* < 0.001). (**b**) The blue line shows the drug persistence rates of ulcerative colitis (UC) patients and the green line shows the drug persistence rates for Crohn’s disease (CD) patients receiving adalimumab. (**c**) The blue line shows the drug persistence rates for UC patients and the green line shows the drug persistence rates for CD patients receiving infliximab. There was no statistically significant difference in the drug persistence rate between UC and CD patients, nor between those receiving adalimumab or infliximab (*p* = 0.403 and *p* = 0.151, respectively).

The results of the Kaplan–Meier survival analysis for evaluation of TP in patients with IBD, comparing biologics and disease types are shown in Figure 1. The TP rate was significantly lower in patients receiving adalimumab than receiving infliximab (mean survival time: 74.3 vs. 99.5 months, *p* < 0.001). However, there was no statistically significant difference in the TP rate between UC (*p* = 0.403) and CD patients (*p* = 0.151).

## 4. Discussion

The use of anti-TNF-α agents has been increasing, with adalimumab and infliximab being the most widely preferred first-line therapies. However, a significant proportion of patients experience sLOR during anti-TNF-α maintenance therapy. The annual risk of sLOR ranges from 10% to 20%, and cumulative rates have been reported to reach as high as 60% over the course of treatment [4,5,6,7]. When sLOR occurs, clinicians are often required to make critical adjustments to the treatment plan. Beyond regulatory recommendations, clinicians commonly perform dose escalation as a strategy to achieve and maintain a response. Several meta-analyses have compared the effects of adalimumab and infliximab dose escalation. However, no previous study has directly compared these two therapies in IBD patients who needed dose escalation following failure of their first anti-TNF-α therapy. Therefore, we evaluated the outcomes of dose escalation therapy in patients who experienced treatment failure with adalimumab or infliximab as first-line biologic agents.

The analysis showed that the dose escalation rate was 35.7% (34.7% vs. 37.0% in the adalimumab and infliximab groups, respectively) in the current study population. The TP rate in IBD patients receiving adalimumab was 35.2%, with a sustained remission rate of 2.1%, compared with 53.2% TP with a 7.1% sustained remission rate in the infliximab group. The r-fTD rate was 62.7% in the adalimumab group and 37.7% in the infliximab group. The pLOR and sLOR rates were significantly higher in the adalimumab group compared with the infliximab group (7.7% vs. 1.6% and 51.4% vs. 28.6%, respectively). While lack of response was similar between the groups, patient intolerance and serious adverse events were less prevalent in the adalimumab group compared with the infliximab group (1.4% vs. 7.9% and 2.1% vs. 7.9%).

In our study, the need for dose escalation for adalimumab and infliximab in IBD patients was comparable to the results of the meta-analysis published by Qiu et al., [4]. The majority (76%) of both treatment groups consisted of CD patients. In contrast, a study by O’Donnell S. et al. reported a 52% dose escalation rate, with a higher prevalence among UC patients compared with CD patients (67% vs. 46%) [20]. The lower dose escalation rate observed in UC patients compared with CD patients in our study may be attributed to reporting total dose escalation rates over an extended follow-up period, rather than annual rates. This suggests that CD patients might require more frequent dose escalations over the long term. Another reason may be differences in the biological history of patients between the studies. Adalimumab and infliximab dose escalation was considered separately in our patients with UC and CD. Among our UC patients, the need for dose escalation was detected in 31.4% of patients in the adalimumab group and 26.5% in the infliximab group. The annual dose escalation rates reported in a recent meta-analysis were 21% for adalimumab and 14% for infliximab [21]. In another study, it was reported that 24% of UC patients treated with adalimumab and 35% of UC patients treated with infliximab required an annual dose escalation [22]. In our CD patients, we found that the dose escalation rates were 35.9% in the adalimumab group and 42.3% in the infliximab group. Ma C. reported similar sLOR rates in CD patients, with 46% in the adalimumab group and 54% in the infliximab group. However, all patients treated with infliximab were biologic-naïve, while 62% of patients treated with adalimumab had prior exposure to biologics [7]. We believe that the differences between our findings and recent studies may stem from the substantial heterogeneity in previous studies, such as variations in dosing regimens, biologic experience, and follow-up duration. The fact that all of our patients were biologic-naïve and received standardized dose escalation regimens with standardized duration intervals contributed to the homogeneity of our study.

In a previous meta-analysis, the overall efficacy of adalimumab dose escalation in UC patients was reported to be 52%, compared with 72% for infliximab. However, investigation of markers of loss of response could not be reliably performed [21]. Another large study reported that 39% of UC patients treated with adalimumab, infliximab, or vedolizumab required dose escalation within 12 months, but reasons for discontinuation were not evaluated [22]. Our study showed that among our UC patients, the pLOR rate was 17.6% in the adalimumab group and 6.7% in the infliximab group; the sLOR was 41.2% in the adalimumab group and 26.7% in the infliximab group. Despite these differences, treatment persistence was statistically similar between patients receiving adalimumab and infliximab. To date, data on predictive factors of r-fTD in UC patients are lacking. In the analysis of predictive factors of r-fTD in our UC patients, we could not identify any factor in either treatment group. The ODESSA-UC study by Ghosh S. et al. reported that infection, sepsis, and IBD-related hospitalization rates in UC patients following dose escalation were below 5% across all groups [23]. The relatively higher rate of severe adverse events in the infliximab group (6.7%) may be attributed to the inclusion of patients exposed to infliximab dose escalation for more than 12 months, along with the reporting of total follow-up data.

In real-world data reported from Hungary, the TP rates with dose escalation of anti-TNF-α agents in CD patients in the first year were 63%, 72%, and 75% for adalimumab, originator infliximab, and biosimilar infliximab, respectively. By the third year, this rate had dropped to 52% for adalimumab and 38% for infliximab. In patients treated with adalimumab, TP was influenced by prior steroid use before the initiation of biologic therapy, while in those treated with infliximab, the need for dose escalation affected sustainability, as did gender. It was not reported that anti-TNF-α therapy was the first-line treatment modality for all patients in that study, suggesting that the data may be relatively heterogeneous [5]. To date, there remain insufficient data on predictive factors of r-fTD in CD patients. We evaluated the outcomes of patients treated with anti-TNF-α agents who underwent dose escalation under a standardized dosing regimen within a specific interval. The pLOR observed in CD patients treated with adalimumab was 4.6% and with infliximab was 0%, while sLOR occurred in 54.6% of patients treated with adalimumab and 29.2% of patients treated with infliximab. Regarding the predictive factors of r-fTD analysis in CD patients treated with adalimumab, a history of previous methotrexate use increased the risk of r-fTD by 1.73 times, steroid dependence by 4.32 times, and concurrent steroid use by 5.37 times. In the infliximab group, family history increased the risk of r-fTD by 2.45 times, and previous methotrexate treatment by 2.15 times. The risk of severe adverse events in CD patients was similar to that in the findings for UC patients.

Kapizioni C. et al. analyzed drug survival in a large cohort of IBD patients treated with biological agents. They found that infliximab had a superior survival rate in IBD patients, free from treatment discontinuation or failure, compared with adalimumab [24]. They did not separately analyze drug survival for dose escalation, despite the fact that they included drug survival in their analysis. The current study showed that those requiring dose escalation under adalimumab treatment had a shorter drug survival time—about 25 months less—compared with those requiring dose escalation with infliximab. Even though adalimumab patients had a lower drug survival rate, this difference was not statistically significant when UC and CD patients were analyzed separately. These findings offer valuable insights for clinicians making decisions about anti-TNF-α dose escalation in IBD patients. However, the question remains: should patients who are sLOR to a first-line anti-TNF-α agent switch to a different anti-TNF-α agent or undergo dose escalation? Although our study did not compare this aspect, Gil-Candel et al. evaluated drug survival outcomes in patients who switched from infliximab or adalimumab as first-line treatment to a second-line anti-TNF agent [25]. In their study, drug survival with second-line adalimumab was 23 months, whereas it was 12.5 months with second-line IFX, although the sample sizes in that study were relatively small. Although larger comparative studies are needed, the current evidence suggests that in patients who experience sLOR to first-line adalimumab or infliximab, dose escalation may provide greater benefits than switching to another anti-TNF-α agent.

Our study had some limitations. Firstly, it was a retrospective study conducted at a single institution. Secondly, we were unable to measure antibody levels during follow-up, preventing us from evaluating this as a potential factor influencing r-fTD. Future studies should investigate the efficacy of dose escalation with other first-line anti-TNF-α therapies, as well as the effectiveness of switching to another biologic agent in patients unresponsive to anti-TNF-α therapy.

## 5. Conclusions

Infliximab dose escalation proved to be more effective than adalimumab dose escalation in IBD patients, while the side effect profile indicated that adalimumab dose escalation was safer than infliximab.

## Figures and Tables

**Table 1:** Demographic, clinical, laboratory data of the IBD patients receiving escalated ADA and IFX therapies.

Characteristics	Total (n = 268)	ADA (n = 142)	IFX (n = 126)	*p*
**Age at onset of IBD (years), median (IQR)**	29.0 (23.0–39.0)	29.0 (23.0–35.2)	30.0 (23.0–40.0)	0.278 ^a^
**Current age (years), median (IQR)**	39.0 (29.0–50.0)	37.0 (30.0–48.0)	40.0 (29.0–53.2)	0.194 ^a^
**Gender, n (%)**				
Male	167 (62.3)	90 (63.4)	77 (61.1)	0.702 ^b^
Female	101 (37.7)	52 (36.6)	49 (38.9)	
**Smoking, n (%)**				
Never	114 (42.5)	62 (43.7)	52 (41.3)	0.894 ^b^
Ex-smoker	87 (32.5)	46 (32.4)	41 (32.5)	
Current smoker	67 (25.0)	34 (23.9)	33 (26.2)	
**Family history of IBD, n (%)**	28 (10.4)	13 (9.2)	15 (11.9)	0.463 ^b^
**BMI (kg/m^2^), median (IQR)**	23.3 (22.9–24.7)	23.3 (23.0–25.0)	23.1 (22.8–24.5)	**0.021 ^a^**
**Total disease duration (years), median (IQR)**	7.5 (3.0–13.0)	8.0 (3.0–12.0)	7.0 (3.0–13.2)	0.916 ^a^
**Time from IBD onset to biologic therapy (months), median (IQR)**	43.2 (13.0–99.7)	42.0 (16.5–89.6)	47.8 (10.2–107.4)	0.953 ^a^
**Follow-up from biologic therapy to dose escalation (months), median (IQR)**	14.7 (7.0–29.6)	12.5 (6.2–28.6)	15.4 (8.4–30.8)	0.280 ^a^
**Follow-up from dose escalation to last visit (months), median (IQR)**	39.7 (19.6–76.2)	38.6 (19.1–81.0)	41.9 (19.6–67.0)	0.816 ^a^
**Disease type, n (%)**				
UC	64 (23.9)	34 (23.9)	30 (23.8)	0.979 ^b^
CD	204 (76.1)	108 (76.1)	96 (76.2)	
**Disease extension in UC patients, n (%)**				
Proctitis	1 (1.6)	1 (2.9)	0 (0.0)	0.890 ^c^
Left-sided colitis	20 (31.3)	11 (32.4)	9 (30.0)	
Extensive colitis	43 (67.2)	22 (64.7)	21 (70.0)	
**Disease location in CD patients, n (%)**				
Ileal (L1)	84 (41.2)	42 (38.9)	42 (43.8)	0.780 ^b^
Colonic (L2)	31 (15.2)	17 (15.7)	14 (14.6)	
Ileo-colonic (L3)	89 (43.6)	49 (45.4)	40 (41.7)	
**Disease behavior in CD patients, n (%)**				
Inflammatory disease (B1)	100 (49.0)	59 (54.7)	41 (42.7)	0.219 ^b^
Stricturing (B2)	51 (25.0)	25 (23.1)	26 (27.1)	
Penetrating (B3)	53 (26.0)	24 (22.2)	29 (30.2)	
**Perianal disease in CD patients, n (%)**	89 (43.6)	47 (43.5)	42 (43.8)	0.973 ^b^
**Appendectomy, n (%)**	22 (8.2)	12 (8.5)	10 (7.9)	0.878 ^b^
**Extra-intestinal manifestations, n (%)**	151 (56.3)	83 (58.5)	68 (54.0)	0.460 ^b^
Peripheral arthralgia, n (%)	101 (37.7)	52 (36.6)	49 (38.9)	0.702 ^b^
Backache, n (%)	51 (19.0)	30 (21.1)	21 (16.7)	0.353 ^b^
Aphthous ulcer, n (%)	42 (15.7)	28 (19.7)	14 (11.1)	0.053 ^b^
Peripheral arthritis, n (%)	35 (13.1)	19 (13.4)	16 (12.7)	0.869 ^b^
Ankylosing spondylitis, n (%)	28 (10.4)	17 (12.0)	11 (8.7)	0.387 ^b^
Sacroiliitis, n (%)	14 (5.2)	8 (5.6)	6 (4.8)	0.749 ^b^
Thromboembolism, n (%)	10 (4.1)	6 (4.2)	5 (4.0)	0.916 ^b^
Erythema nodosum, n (%)	9 (3.4)	8 (5.6)	1 (0.8)	**0.039 ^c^**
PSC, n (%)	4 (1.5)	2 (1.4)	2 (1.6)	0.999 ^c^
Pyoderma gangrenous, n (%)	3 (1.1)	3 (2.1)	0 (0.0)	0.250 ^c^
Uveitis, n (%)	3 (1.1)	3 (2.1)	0 (0.0)	0.250 ^c^
Others, n (%)	4 (1.5)	1 (0.7)	3 (2.4)	0.345 ^c^
**Prior major abdominal surgery for IBD, n (%)**	111 (41.4)	58 (40.8)	53 (42.1)	0.840 ^b^
**Prior conventional medications, n (%)**	243 (90.7)	132 (93.0)	111 (88.1)	0.172 ^b^
Mesalazine oral, n (%)	214 (79.9)	112 (78.9)	102 (81.0)	0.672 ^b^
Mesalazine enema, n (%)	59 (22.0)	35 (24.6)	24 (19.0)	0.269 ^b^
Mesalazine suppository, n (%)	11 (4.1)	7 (4.9)	4 (3.2)	0.470 ^b^
Sulfasalazine, n (%)	48 (17.9)	25 (17.6)	23 (18.3)	0.890 ^b^
Budesonide, n (%)	55 (20.5)	40 (28.2)	15 (11.9)	**0.001 ^b^**
Steroids, n (%)	191 (71.3)	104 (73.2)	87 (69.0)	0.449 ^b^
Thiopurine, n (%)	238 (88.8)	125 (88.0)	113 (89.7)	0.668 ^b^
Methotrexate, n (%)	96 (35.8)	56 (39.4)	40 (31.7)	0.190 ^b^
**Steroid dependence, n (%)**	249 (92.9)	129 (90.8)	120 (95.2)	0.162 ^b^
**Concomitant medication at biologic initiation, n (%)**	122 (45.5)	63 (44.4)	59 (46.8)	0.687 ^b^
Immunomodulatory therapy ^d^	119 (44.4)	60 (42.3)	59 (46.8)	0.452 ^b^
Thiopurine	82 (30.6)	36 (25.4)	46 (36.5)	**0.048 ^b^**
Methotrexate	37 (13.8)	24 (16.9)	13 (10.3)	0.119 ^b^
Mesalazine	4 (1.5)	4 (2.8)	0 (0.0)	0.125 ^c^
Sulfasalazine	0 (0.0)	0 (0.0)	0 (0.0)	n.a.
Budesonide	0 (0.0)	0 (0.0)	0 (0.0)	n.a.
Steroids	7 (2.6)	6 (4.2)	1 (0.8)	0.125 ^c^
**Baseline CDAI, median (IQR)**	335.0 (248.5-423.0)	347.5 (256.0–431.8)	319.5 (225.8–415.5)	0.230 ^a^
**Baseline partial Mayo score, median (IQR)**	6.0 (5.0–7.3)	6.0 (5.0–8.0)	7.0 (6.0–7.0)	0.590 ^a^
**Baseline endoscopic Mayo score, median (IQR)**	2.0 (2.0–3.0)	3.0 (2.0–3.0)	2.0 (2.0–3.0)	0.172 ^a^
**Baseline CRP (mg/L), median (IQR)**	6.0 (5.0–22.5)	5.9 (4.2–22.1)	6.5 (5.0–24.3)	0.237 ^a^
**Baseline Hb (g/dL), median (IQR)**	12.1 (11.6–13.7)	12.2 (11.8–13.9)	12.0 (11.4–13.1)	0.052 ^a^
**Baseline Albumin (g/dL), median (IQR)**	4.0 (3.9–4.3)	4.0 (3.9–4.3)	4.0 (3.9–4.3)	0.715 ^a^

^a^ Mann–Whitney U test was used. ^b^ Pearson Chi-square test was used. ^c^ Fisher’s exact test was used. ^d^ Immunomodulatory therapy included thiopurine and methotrexate treatments. Note: IBD: inflammatory bowel disease, ADA: adalimumab, IFX: infliximab, IQR: interquartile range, BMI: body mass index, UC: ulcerative colitis, CD: Crohn’s disease, PSC: primary sclerosing cholangitis, CDAI: Crohn’s disease activity index, CRP: C-reactive protein, Hb: hemoglobin, n.a.: not applicable. Statistically significant values (*p* ≤ 0.05) are given in bold.

**Table 2:** Analysis of the treatment outcomes in IBD patients receiving ADA and IFX dose escalation therapies.

	Total (n = 268)	ADA (n = 142)	IFX (n = 126)	*p*
**Treatment persistence, n (%)**	117 (43.7)	50 (35.2)	67 (53.2)	**0.003 ^a^**
**Treatment discontinuation, n (%)**	151 (56.3)	92 (64.8)	59 (46.8)	
**Sustained remission, n (%)**	12 (4.5)	3 (2.1)	9 (7.1)	0.047 ^a^
**Remission-free treatment discontinuation, n (%)**	139 (51.9)	89 (62.7)	50 (39.7)	<0.001 **^a^**
**Patient lack of compliance, n (%)**	4 (1.5)	2 (1.4)	2 (1.6)	0.999 ^b^
**Patient intolerance, n (%)**	12 (4.5)	2 (1.4)	10 (7.9)	**0.010 ^a^**
**Therapeutic failure, n (%)**	122 (45.5)	84 (59.1)	38 (30.2)	**<0.001 ^a^**
**Primary lack of response, n (%)**	13 (4.9)	11 (7.7)	2 (1.6)	**0.019 ^a^**
**Secondary loss of response, n (%)**	109 (40.7)	73 (51.4)	36 (28.6)	**<0.001 ^a^**
Steroid, n (%)	19 (7.1)	15 (10.6)	4 (3.2)	**0.019 ^a^**
Hospitalization, n (%)	15 (5.6)	9 (6.3)	6 (4.8)	0.575 ^a^
Surgery, n (%)	10 (3.7)	6 (4.2)	4 (3.2)	0.754 ^b^
Switch to another biotherapy, n (%)	107 (39.9)	72 (50.7)	35 (27.8)	**<0.001 ^a^**
Serious adverse events, n (%)	13 (4.9)	3 (2.1)	10 (7.9)	**0.027 ^a^**
Allergic reaction, n (%)	7 (2.6)	0 (0.0)	7 (5.6)	**0.005 ^b^**
Serious infection, n (%)	3 (1.1)	3 (2.1)	0 (0.0)	0.250 ^b^
Other serious adverse events, n (%)	3 (1.1)	0 (0.0)	3 (2.4)	0.103 ^b^

^a^: The Pearson Chi-square test was used; ^b^: Fisher’s exact test was used. Note: IBD: inflammatory bowel disease, ADA: adalimumab, IFX: infliximab, n.a.: not applicable. Statistically significant values (*p* ≤ 0.05) are given in bold.

**Table 3:** Subgroup analysis of treatment outcomes in IBD patients receiving ADA and IFX dose escalation therapies.

Characteristics	ADA		*p*	IFX		*p*
	UC (n = 34)	CD (n = 108)		UC (n = 30)	CD (n = 96)	
**Treatment persistence, n (%)**	11 (32.4)	39 (36.1)	0.689 ^a^	15 (50.0)	52 (54.2)	0.690 ^a^
**Treatment discontinuation, n (%)**	23 (67.6)	69 (63.9)		15 (50.0)	44 (45.8)	
**Sustained remission, n (%)**	1 (2.9)	2 (1.9)	0.563 ^b^	2 (6.7)	7 (7.3)	0.999 ^b^
**Remission-free treatment discontinuation, n (%)**	22 (64.7)	67 (62.0)	0.779 ^a^	13 (43.3)	37 (38.5)	0.640 ^a^
**Patient lack of compliance, n (%)**	1 (2.9)	1 (0.9)	0.423 ^b^	1 (3.3)	1 (1.0)	0.421 ^b^
**Patient intolerance, n (%)**	1 (2.9)	1 (0.9)	0.423 ^b^	2 (6.7)	8 (8.3)	0.999 ^b^
**Therapeutic failure, n (%)**	20 (58.8)	64 (59.3)	0.964 ^a^	10 (33.3)	28 (29.2)	0.664 ^a^
**Primary lack of response, n (%)**	6 (17.6)	5 (4.6)	**0.023 ^b^**	2 (6.7)	0 (0.0)	0.055 ^b^
**Secondary loss of response, n (%)**	14 (41.2)	59 (54.6)	0.171 ^a^	8 (26.7)	28 (29.2)	0.791 ^a^
Steroid, n (%)	6 (17.6)	9 (8.3)	0.199 ^b^	1 (3.3)	3 (3.1)	0.999 ^b^
Hospitalization, n (%)	2 (5.9)	7 (6.5)	0.999 ^b^	0 (0.0)	6 (6.3)	0.334 ^b^
Surgery, n (%)	2 (5.9)	4 (3.7)	0.630 ^b^	2 (6.7)	2 (2.1)	0.240 ^b^
Switch to another biotherapy, n (%)	14 (41.2)	58 (53.7)	0.563 ^b^	8 (26.7)	27 (28.1)	0.563 ^b^
**Serious adverse events, n (%)**	1 (2.9)	2 (1.9)	0.563 ^b^	2 (6.7)	8 (8.3)	0.999 ^b^
Allergic reaction, n (%)	0 (0.0)	0 (0.0)	n.a.	1 (3.3)	6 (6.3)	0.999 ^b^
Serious infection, n (%)	1 (2.9)	2 (1.9)	0.563 ^b^	0 (0.0)	0 (0.0)	n.a.
Other serious adverse events, n (%)	0 (0.0)	0 (0.0)	n.a.	1 (3.3)	3 (2.1)	0.561 ^b^

^a^: The Pearson Chi-square test was used; ^b^: Fisher’s exact test was used. Note: ADA: adalimumab, IFX: infliximab, UC: ulcerative colitis, CD: Crohn’s disease, n.a.: not applicable. Statistically significant values (*p* ≤ 0.05) are given in bold.

**Table 4:** Bivariate and multivariate analysis of predictors of discontinuation in IBD patients receiving escalated ADA and IFX therapies.

Characteristics	Total (n = 268)			ADA (n = 142)			IFX (n = 126)		
	Bivariate Analysis	Multivariate Analysis		Bivariate Analysis	Multivariate Analysis		Bivariate Analysis	Multivariate Analysis	
	*p*	HR (95 CI%)	*p*	*p*	HR (95 CI%)	*p*	*p*	HR (95 CI%)	*p*
**Age at onset of IBD (years)**	0.606			0.767			0.823		
**Current age (years)**	0.219			0.190			0.826		
**Female gender**	0.793			0.830			0.803		
**Smoking**									
Never		1			1				
Ex-smoker	0.052	0.64 (0.43–0.94)	**0.024**	0.061	0.58 (0.35–0.96)	**0.033**	0.420		
Current smoker	0.354	1.06 (0.69–1.64)	0.793	0.128	0.75 (0.43–1.31)	0.315	0.621		
**Family history of IBD**	0.054	1.01 (0.59–1.76)	0.960	0.909			**0.006**	2.34 (1.20–4.57)	**0.012**
**BMI (kg/m^2^)**	0.724			0.946			0.858		
**Total disease duration (years)**	0.133			**0.032**	0.97 (0.93–1.02)	0.183	0.981		
**Appendectomy**	0.791			0.447			0.521		
**Prior major abdominal surgery for IBD**	0.071	1.85 (1.29–2.67)	**0.001**	0.518			0.055	2.71 (1.37–5.35)	**0.004**
**Prior conventional medications**	0.626			0.167			0.162		
Mesalazine oral	0.117			0.464			0.103		
Mesalazine enema	**0.003**	2.42 (1.59–3.66)	**<0.001**	0.084	1.49 (0.93–2.38)	0.101	**0.008**	4.16 (1.90–9.12)	**<0.001**
Mesalazine suppository	0.667			0.336			0.736		
Sulfasalazine	0.444			0.254			0.876		
Budesonide	0.552			0.707			0.349		
Steroids	0.156			0.149			0.870		
Thiopurine	0.666			0.210			0.399		
Methotrexate	**0.001**	1.83 (1.29–2.59)	**0.001**	**0.005**	1.75 (1.10–2.77)	**0.018**	0.155		
**Steroid dependence**	0.067	1.86 (0.86–4.02)	0.117	**0.014**	3.90 (1.20–12.68)	**0.023**	0.378		
**Concomitant medication at biologic initiation**	0.374			0.539			0.087		
Immunomodulatory therapy ^a^	0.378			0.441			0.087 ^a^	0.79 (0.44–1.40)	0.413
Thiopurine	0.462			0.551			0.318		
Methotrexate	0.760			0.713			0.245		
Mesalazine	0.930			0.686			n.a.		
Steroids	**<0.001**	5.31 (2.22–12.68)	**<0.001**	**<0.001**	4.01 (1.61–9.93)	**0.003**	0.106		
**Baseline CRP (mg/L)**	0.176			0.342			0.216		
**Baseline Hb (** **g/dL)**	0.398			0.159			0.843		
**Baseline Albumin (g/dL)**	0.139			0.079	0.64 (0.40–1.04)	0.069	0.558		

^a^: Concomitant immunomodulatory therapy was included in the multivariate model instead of concomitant medication, due to the high correlation between these two variables. Note: IBD: inflammatory bowel disease, ADA: adalimumab, IFX: infliximab, HR: hazard ratio, CI: confidence interval, BMI: body mass index, CRP: C-reactive protein, Hb: hemoglobin, n.a.: not applicable. Statistically significant values (*p* ≤ 0.05) are given in bold.

**Table 5:** Bivariate and multivariate analysis of predictors of discontinuation among UC and CD patients receiving escalated ADA and IFX therapies.

Characteristics	ADA (n = 142)						IFX (n = 126)					
	UC (n = 34)			CD (n = 108)			UC (n = 30)			CD (n = 96)		
	Bivariate Analysis	Multivariate Analysis		Bivariate Analysis	Multivariate Analysis		Bivariate Analysis	Multivariate Analysis		Bivariate Analysis	Multivariate Analysis	
	*p*	HR (95 CI%)	*p*	*p*	HR (95 CI%)	*p*	*p*	HR (95 CI%)	*p*	*p*	HR (95 CI%)	*p*
**Age at onset of IBD (years)**	0.904			0.814			0.129			0.640		
**Current age (years)**	0.325			0.350			0.068	0.97 (0.93–3.03)	0.127	0.686		
**Female gender**	0.446			0.989			0.759			0.630		
**Smoking**												
Never												
Ex-smoker	0.185			0.163			0.492			0.726		
Current smoker	0.915			0.259			0.317			0.203		
**Family history of IBD**	0.592			0.463			0.551			**0.003**	2.45 (1.11–5.42)	**0.027**
**BMI (kg/m^2^)**	0.057	0.88 (0.73–1.06)	0.172	0.523			0.636			0.665		
**Total disease duration (years)**	0.106			0.112			0.469			0.948		
**Disease extension in UC patients**												
Proctitis (E1)												
Left-sided colitis (E2)	0.311						0.906					
Extensive colitis (E3)	0.781						n.a.					
**Disease location in CD patients**												
Ileal (L1)												
Colonic (L2)				0.947						0.502		
Ileo-colonic (L3)				0.117						0.826		
**Disease behavior in CD patients**												
Inflammatory disease (B1)											1	
Stricturing (B2)				0.817						0.605	0.78 (0.14–4.40)	0.774
Penetrating (B3)				0.815						**0.004**	1.41 (0.23–6.62)	0.711
**Perianal disease in CD patients**				0.255						0.209		
**Appendectomy**	0.137			0.339			n.a.			0.311		
**Prior major abdominal surgery for IBD**	0.053	1.42 (0.45–4.46)	0.551	0.887			0.361			**0.021**	1.70 (0.33–8.85)	0.527
**Prior conventional medications**	0.393			0.250			n.a.			0.067		
Mesalazine oral	0.393			0.165			0.448			0.053	0.50 (0.24–1.06)	0.072
Mesalazine enema	0.176			0.592			0.129			0.333		
Mesalazine suppository	0.729			0.272			0.305			0.282		
Sulfasalazine	0.383			0.076	1.47 (0.82–2.62)	0.193	**0.006**	5.46 (1.44–20.66)	**0.013**	0.390		
Budesonide	0.616			0.920			n.a.			0.177		
Steroids	0.976			0.163			0.597			0.616		
Thiopurine	0.886			0.136			0.880			0.241		
Methotrexate	0.371			**0.003**	1.73 (1.04–2.87)	**0.035**	0.766			**0.035**	2.15 (1.12–4.15)	**0.022**
**Steroid dependence**	n.a.			**0.017**	4.32 (1.34–13.91)	**0.014**	0.570			0.437		
**Concomitant medication at biologic initiation**	0.645			0.393			0.653			0.081		
Immunomodulatory therapy ^a^	0.550			0.262			0.653			0.081	0.79 (0.40–1.55)	0.495
Thiopurine	0.470			0.315			0.109			0.149		
Methotrexate	0.832			0.652			0.378			0.609		
Mesalazine	0.697			0.573			n.a.			n.a.		
Steroids	0.098	1.50 (0.13–17.82)	0.749	**<0.001**	5.37 (1.77–16.36)	**0.003**	n.a.			0.079	1.22 (0.11–13.37)	0.869
**Baseline CDAI**				0.539						**0.017**	1.00 (0.99–1.01)	0.164
**Baseline partial Mayo score**	**0.007**	1.46 (0.95–2.23)	0.084				0.191					
**Baseline endoscopic Mayo score**	0.619						0.796					
**Baseline CRP (mg/L)**	**0.014**	1.01 (0.97–1.04)	0.697	0.553			0.345			0.192		
**Baseline Hb (g/dL)**	0.118			0.384			0.393			0.926		
**Baseline albumin (g/dL)**	0.477			**0.036**	0.59 (0.35–1.02)	0.058	0.111			0.453		

^a^ Concomitant immunomodulatory therapy was included in the multivariate model instead of concomitant medication due to the high correlation between these two variables. Note: UC: ulcerative colitis, CD: Crohn’s disease, ADA: adalimumab, IFX: infliximab, HR: hazard ratio, CI: confidence interval, IBD: Inflammatory bowel disease, BMI: body mass index, CDAI: Crohn’s disease activity index, CRP: C-reactive protein, Hb: hemoglobin, n.a.: not applicable. Statistically significant values (*p* ≤ 0.05) are given in bold.

## Data Availability

The data used and analyzed in the current study are available from the corresponding author upon reasonable request.

## Supplementary Materials

The following supporting information can be downloaded at: https://www.mdpi.com/article/10.3390/jcm14041228/s1, Figure S1: UC: ulcerative colitis, CD: Crohn’s disease, IC: indeterminate colitis, ADA: adalimumab, IFX: infliximab.
