# Supplementary material for: Comparative Outcomes of Adalimumab and Infliximab Dose Escalation in Inflammatory Bowel Disease Patients Failing First-Line Biologic Treatment
Source: J Clin Med. 2025 Feb 13;14(4):1228. doi: 10.3390/jcm14041228 (PMC11857063; doi:10.3390/jcm14041228)
Supplement: Supplementary file 1 [file jcm-14-01228-s001.zip › jcm-3439908-supplementary.pdf]

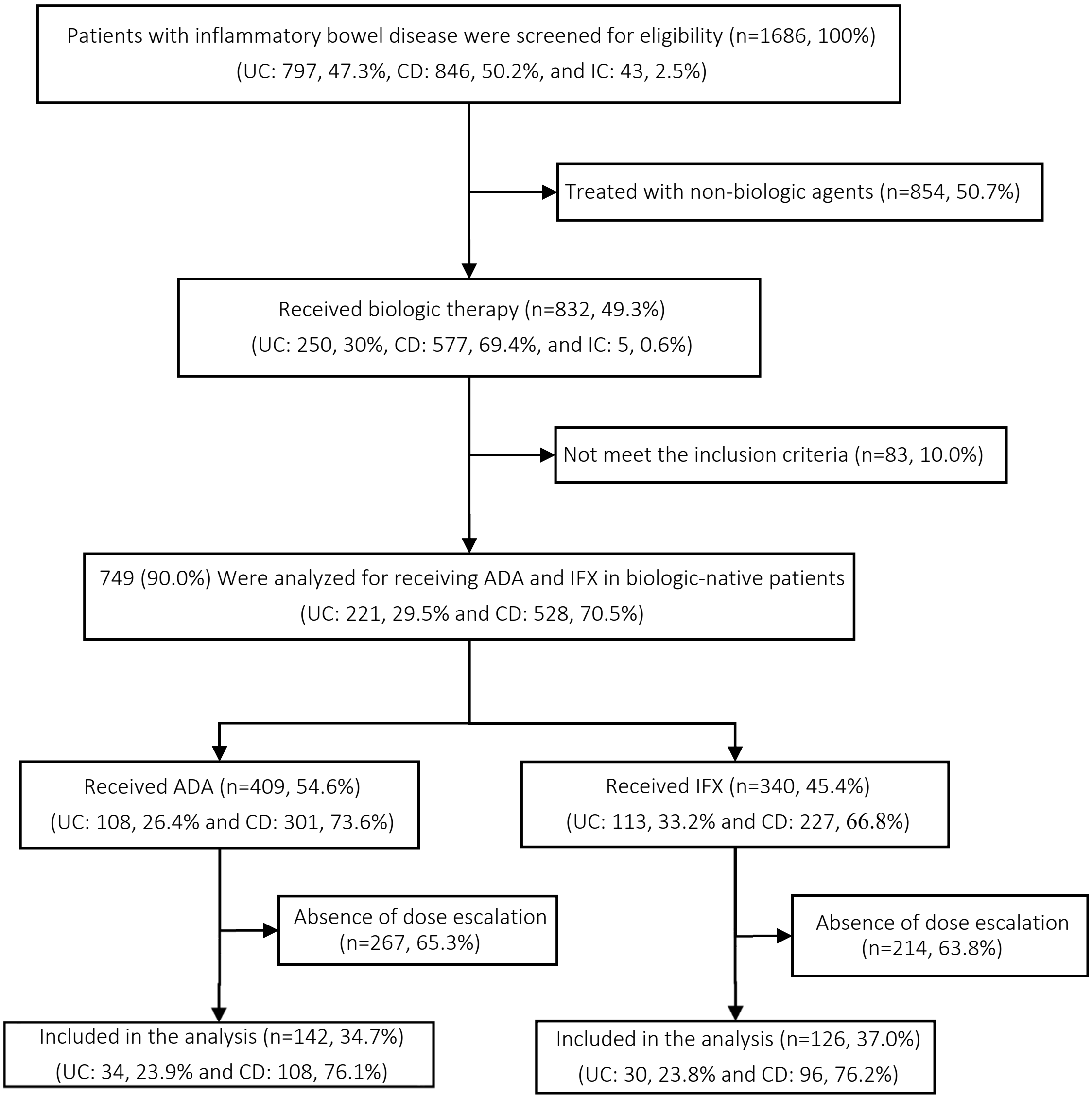

Figure S1. UC: ulcerative colitis, CD: Crohn's disease, IC: indeterminate colitis, ADA: adalimumab, IFX: infliximab.
